# Unintended pregnancy and contraceptive use among women in low- and middle-income countries: systematic review and meta-analysis

**DOI:** 10.1186/s40834-023-00255-7

**Published:** 2023-11-23

**Authors:** Kelemu Abebe Gelaw, Yibeltal Assefa Atalay, Natnael Atnafu Gebeyehu

**Affiliations:** 1 School of Midwifery, College of Medicine and Health Sciences, Wolaita Sodo University, Wolaita Sodo, Ethiopia; 2 School of Public Health, College of Health Science and Medicine, Wolaita Sodo University, Wolaita Sodo, Ethiopia

**Keywords:** Unintended pregnancy, Unwanted pregnancy, Contraceptive use, Low- and middle-income countries

## Abstract

**Introduction:**

Unintended pregnancy is a leading cause of maternal mortality associated with abortion, inadequate contraceptive use, contraceptive failure, and contraceptive discontinuation in low- and middle-income countries. Most unintended pregnancies occur in regions with limited availability of maternal health services, resulting in a significant number of maternal deaths. Therefore, this review aimed to assess the overall prevalence of unintended pregnancy among women using contraceptives in low- and middle-income countries.

**Method:**

PubMed, Science Direct, Google Scholar, Scopus, and the Ethiopian University Online Library were searched. Data were extracted using Microsoft Excel and analyzed using STATA statistical software (version 14). Publication bias was checked using forest plot, Begg rank test, and Egger regression test. To check for heterogeneity, I^2^ was calculated and an overall estimation analysis was performed. Subgroup analysis was conducted by study setting, study design, and publication. The Joanna Briggs Institute quality assessment tool was used to assess the quality of each study. We performed a one-time sensitivity analysis.

**Results:**

Of the 1304 articles retrieved, 23 studies (involving 40,338 subjects) met the eligibility criteria and were included in this study. The pooled prevalence of unintended pregnancy among women using contraceptives in low- and middle-income countries was 44.68% (95% CI: 35.16–54.20; I2 = 99.7%, *P* < 0.001). Based on subgroup analysis, the pooled prevalence of unintended events was 43.58% (CI: 32.99, 54.173) and 49.93% (CI: 28.298, 71.555) for cross-sectional and cohort studies, respectively. Based on the study design, it was 34.47% (CI: 27.012, 41.933) for community studies and 55.85% (CI: 33.364, 78.339) for institutional studies.

**Conclusion:**

The overall prevalence of unintended pregnancy was high among women using contraceptives in low- and middle-income countries. Therefore, it is better to pay attention to prevention strategies for unintended pregnancy, such as information and education accessibility and contraceptive utilization.

**Supplementary Information:**

The online version contains supplementary material available at 10.1186/s40834-023-00255-7.

## Introduction

According to data, approximately 40% of pregnancies in women of childbearing age worldwide are classified as unintended [1]. The World Health Organization (WHO) defines an unwanted pregnancy as a pregnancy that was neither wanted nor planned at the time of conception [2]. Similarly, the International Federation of Obstetricians and Gynecologists (FIGO) defines an unintended pregnancy as one that is either unplanned or mistimed at the time of conception [3].

Unintended pregnancies represent a significant public, clinical, and social health problem worldwide, as they are often associated with abortion and its resulting complications. These complications are often due to inadequate abortion care services, particularly in resource-limited facilities [4]. Available evidence also shows that there are an estimated 80 million unintended pregnancies annually in low- and middle-income countries [5]. This unintended pregnancy is closely associated with an increased likelihood of preterm labor, low birth weight infants, unsafe abortion procedures, and maternal depressive episodes [6, 7].

A significant number of women worldwide do not have adequate access to contraceptives, despite the implementation of some goals [8]. Current estimates suggest that approximately 257 million women worldwide who want to avoid pregnancy are not using safe and modern contraceptive methods. Furthermore, in regions where data are available, almost a quarter of women cannot independently refuse sexual intercourse [9].

In developing countries, providing adequate access to modern contraceptive methods could enable women to prevent an estimated 67 million unintended pregnancies, 23 million unplanned births, 36 million abortions, and 76,000 maternal deaths annually [10]. Furthermore, the lack of such access contributes to the prevalence of unsafe abortions, which are a major contributor to maternal mortality worldwide [11]. An unwanted pregnancy can also lead to an undesirable outcome, namely, the occurrence of adverse consequences such as infant mortality and morbidity. Extensive literature suggests that the main causes of unintended pregnancy are due to ineffective use of contraceptive methods, including cases of incorrect or omitted use of contraceptives, discontinuation of contraceptive practices, and cases of contraceptive failure [12–15].

Several factors were also found to be associated with socio-demographic and economic factors, early initiation of sexual activity, availability of health services, limited access to family planning resources, increased parity, contraceptive failure, partner preference for offspring, and domestic violence phenomenon of unplanned pregnancies [16–18].

The purpose of this study is to assess women's contraceptive practices before pregnancy and whether their encounters with unwanted pregnancies impact their use and choice of contraceptive methods. The aim is to improve the effectiveness of the use of contraceptives in women who have become pregnant unintentionally [19]. It is important to note that low- and middle-income countries (LMICs) have not been the focus of research on these aspects to date. Little research has been done on this topic in LMICs, even though unintended pregnancies can account for up to 43% of all pregnancies [20].

Previous studies have suggested varying rates of unintended pregnancy in low- and middle-income countries (LMICs), with estimates ranging from 5.8% in Congo [21] to 92.24% in Iran [22]. However, due to these inconsistencies, a comprehensive review and meta-analysis examining the prevalence of unintended pregnancy in LMICs is needed. Therefore, there is a need for review to improve the ability to provide updated scientific evidence that can effectively guide the development of policies and programs to improve women's reproductive and sexual health in low- and middle-income countries. Thus, this systematic meta-analysis aimed to assess the overall prevalence of unintended planned pregnancy among women using contraception in low- and middle-income countries.

## Methods

### Search strategy

International online databases (Pub Med, Science Direct, Scopus, and Google Scholar) were used to search for articles on the prevalence of unintended pregnancy among contraceptive users of reproductive-age women. We also retrieved gray literature from Addis Ababa University's online research institutional repository. The search string was established by using "AND" and "OR" Boolean operators. The search strategies for Science Direct, Scopus, and Google Scholar were “prevalence of Mistimed pregnancy; unintended pregnancy; unplanned pregnancy; unwanted pregnancy, and low- and middle-income countries".

PubMed was searched on ((((Contraceptive OR ("Contraceptive" OR "contraception" OR "family planning" OR "contraceptive device" OR "contraceptive agents" OR "birth control device" AND (Unintended pregnancy OR accidental pregnancy)) OR ("Unintended pregnancy " OR "pregnancy, unplanned" OR "Pregnancy, unwanted" OR " pregnancy, mistimed" AND (Low- and middle-income countries OR low-income countries OR middle-income countries OR resource-limited countries OR poor countries OR third-world countries). Searching terms were based on PICO principles to retrieve relevant articles through the aforementioned databases. PICO questions adapted to the “PEO” (population, exposure, and outcome) style. The search period was from February 1/2021 to January 24/2022.

### Reporting

We reported the results according to the PRISMA (Preferred Reporting Items for Systematic Reviews and Meta-analyses) criteria for conducting the systematic review [23] (Supplementary file 1). We checked Prospero to see if any authors had registered this systematic review and meta-analysis work, but none had.

### PEO Guide

**P: Population (Patients)**

- ✓ Women who had unintended pregnancy among contraceptive users in low- and middle-income countries

**E: Exposure**

- ✓ Women who had unintended pregnancies in low- and middle-income countries

**O: Outcome**

- ✓ The prevalence of unintended pregnancy among women who use contraceptives in low- and middle-income countries

### Outcome measurement

Unintended pregnancy: Unintended pregnancies are pregnancies that were either unwanted or mistimed at the time of conception. According to conventional guidelines, both wanted later (mistimed pregnancy) and wanted no more (unwanted pregnancy) are categorized as unintended pregnancies [24–26]. In this study, we examined the phenomenon of unwanted pregnancy and integrated the two different criteria of “later wanted” and “no longer wanted” with the concept of “unwanted pregnancy in women using contraceptives”.

### Eligibility Criteria

#### Inclusion criteria

Only English-language articles (both published and unpublished studies) that were full-text searchable and that were written in low- and middle-income countries were included in this meta-analysis of all studies reporting the prevalence of unintended pregnancy among women of childbearing age. Observational studies (cross-sectional and cohort) reported the prevalence of unintended pregnancy among women of childbearing age as study participants.

#### Exclusion criteria

This systematic review and meta-analysis excluded studies that had duplicate sources, qualitative studies, case reports, case series, opinion pieces, letters, and articles where the full text was not accessible.

### Quality assessment

Using a standardized quality rating checklist developed by the Joanna Briggs Institute (JBI), three authors (KA and YA) independently assessed the studies' quality [27]. Through discussion led by the third author, any disagreements that arose during the quality evaluation were resolved (NA). Finally, a resolution and consensus were reached regarding the argument. The critical analysis checklist has eight parameters with yes, no, unclear, and not applicable options. The parameters involve the following questions:

1. Were the criteria for inclusion in the sample clearly defined?
2. Were the study subjects and therefore the setting described in detail?
3. Was the exposure measured result validly and reliably?
4. Were the main objective and standard criteria used for the measurement of the event?
5. Were confounding factors identified?
6. Were strategies to affect confounding factors stated?
7. Were the results measured truly and dependably?
8. Was the statistical analysis suitable? Studies were considered low risk when they scored 50% and above on the quality assessment indicators as reported in a supplementary file (Supplementary file 2).

### Risk of bias assessment

Using the method described by Hoy et al. Bias assessment tools have been developed [28], consisting of 10 items to assess four dimensions of bias as well as internal and external validity. Two authors (KA and YA) independently assessed the included studies for risk of bias. The third author led a dialogue to resolve any disagreements that arose during the risk of bias (NA) assessment. The debate was tested and consensus was reached. The presence of selection bias, nonresponse bias, and external validity is assessed using the first four items (Items 1–4). The remaining six items (Items 5–10) assess internal validity, measurement-related bias, and analysis-related bias. Studies were classified as “low risk of bias” if they answered “yes” to eight or more of the ten questions. Studies classified as “high risk” were those that received “yes” answers to five or fewer of the ten questions, while studies classified as “medium risk” were those that received “yes” answers on six to seven of the ten questions. -received responses (Supplementary file 3).

### Data extraction

Using a Joanna Briggs Institute standardized data extraction format, two authors (KA and YA) independently extracted all relevant data. A discussion organized by the third author was able to address the conflict that arose during data extraction (NA). The dispute was ultimately resolved and a consensus was reached. The lack of a paper form (manual data) in this study prevented the use of the data automation tool. The first name of each author, year of publication, country of study, setting, research design, incidence of unintended pregnancy, sample size, and quality were all extracted.

### Statistical analysis

Following the extraction of pertinent findings into a Microsoft Excel spreadsheet, the data were subsequently transferred to STATA software version 14 for analysis. To assess the possible presence of publication bias, two methods were used: a funnel plot and Begg and Egger regression tests. A significance level of *P* < 0.05 was used to indicate the possibility of publication bias. In addition, the presence of heterogeneity between studies was assessed using the Cochrane Q statistic. The degree of heterogeneity between studies was quantified using I^2^, with values ​​of 0%, 25%, 50%, and 75% representing no, low, moderate, and high heterogeneity, respectively. To visually assess the presence of heterogeneity, a forest patch was used to represent a forest patch at an elevated level. The analysis used a random-effects model to estimate the overall prevalence of unintended pregnancy. Subgroup analysis was performed based on study setting, study design, and publication status (published vs. unpublished). Additionally, a sensitivity analysis was performed to determine the influence of a single study on the overall prevalence estimate derived from the meta-analysis. The results of the study were presented through text descriptions, tables, and figures.

## Results

### Search findings and study selection

**Fig. 1:**
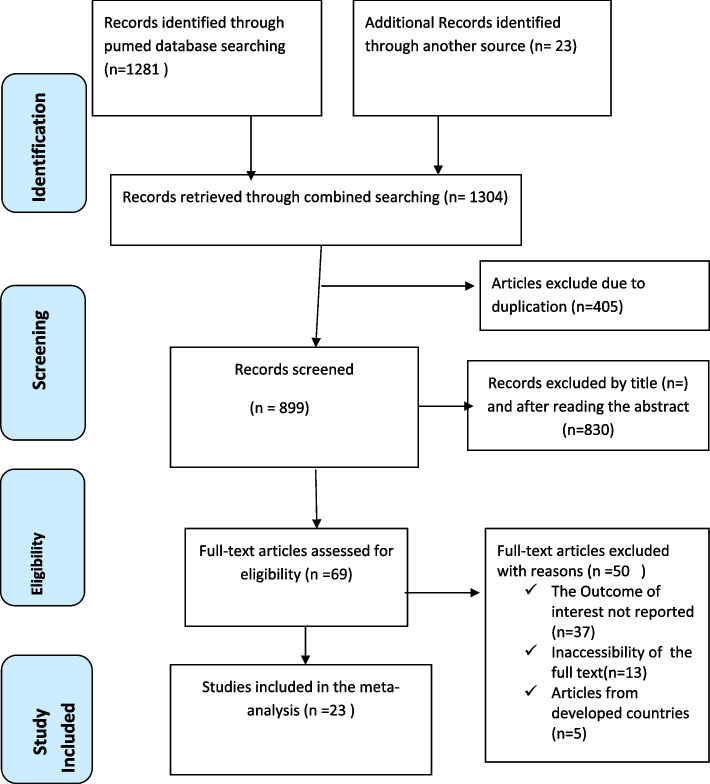
PRISMA flow chart displays the article selection process for systematic review and meta-analysis of unintended pregnancy among contraceptive user women in low and middle-income countries

One thousand three hundred four (1,304) were identified through a comprehensive search of international databases, including Pub Med, Science Direct, Scopus, and Google Scholar. After the initial screening process, 405 articles were identified as duplicates and subsequently removed from the dataset. In addition, 830 studies were excluded after a thorough review of their titles and abstracts. Consequently, 69 articles remained for further evaluation to determine their eligibility for inclusion in the study. A total of 23 studies [21, 22, 26, 29–48] with 40,338 study participants were ultimately included in this systematic review and meta-analysis (Fig. 1).

### Characteristics of Included Studies

Table 1 shows the characteristics of the studies that were included in this analysis. These characteristics include the first author's name, publication year, study setting, study design, sample size, country, and prevalence of unintended pregnancy. A total of 23 studies were included in this analysis. Among these studies, 19 were cross-sectional, while the remaining four were cohort studies. Furthermore, 21 of the studies were published in peer-reviewed journals, while the remaining 2 were unpublished articles.

**Table 1:** Characteristics of the included studies in the systematic review and meta-analysis for the prevalence of unintended pregnancy among contraceptive-user women in low- and middle-income countries

Author	Year	Country	Setting	Study design	Sample size	Prevalence	Quality
Aghababaei S et al. [22]	2017	Iran	Institutional	Cross-sectional	900	92.2	Low-risk
Efrani A [29]	2013	Iran	Community	Cross-sectional	874	21	Low-risk
Fotso JC et al. [30]	2014	Kenya	Community	Cross-sectional	800	23.7	Low-risk
Irina S et al. [31]	Unpub	Moldova	Institution	Cross-sectional	600	82.6	Low-risk
Grindlay K et. al [32]	2018	Ghana	Community	Cross-sectional	350	45	Low-risk
Gomez AM [33]	2011	Colombia	Community	Cross-sectional	4913	62	Low-risk
Marcel Yotebieng et.al [21]	2015	Congo	Institutional	Cross-sectional	699	5.8	Low-risk
Peach E. et. al [34]	2021	Guinea	Institutional	Cross-sectional	699	55	Low-risk
Schaan MM et. al [35]	2014	Botswana	Institutional	Cross-sectional	155	94	Low-risk
Moon TD et. al [36]	2021	Kenya	Community	Cross-sectional	3642	36.7	Low-risk
Hultstrand JN et.al [37]	2019	Switzerland	Institutional	Cross-sectional	1436	70	Low-risk
Jarolimova J. et.al [38]	2018	Uganda	Institutional	Cohort	455	45	Low-risk
Mayondi GK et.al [39]	2016	Botswana	Institutional	Cohort	941	44	Low-risk
Wall KM et. al [40]	2013	Zambia	Institutional	Cohort	137	87	Low-risk
Luchters S et.al [41]	2016	Kenya	Community	Cohort	400	24	Low-risk
McCoy SI et. al [42]	2014	Zimbabwe	Community	cross-sectional	8797	35.1	Low-risk
Joshi B et. al [43]	2015	India	Institutional	Cross-sectional	300	16.6	Low-risk
Omokhodion FO et al. [44]	2017	Nigeria	Community	Cross-sectional	1687	29.8	Low-risk
Chanda MM et. al [45]	2017	Zambia	Community	cross-sectional	945	61	Low-risk
Tiruye et.al	2020	Ethiopia	Community	Cross-sectional	788	26	Low-risk
Nance N, et. al [46]	2018	Zimbabwe	Community	Cross-sectional	10,224	31	Low-risk
Ndifon WO et al. [47]	2006	Nigeria	Institutional	Cross-sectional	195	22.1	Low-risk
Arega T [48]	Unpub	Ethiopia	Community	Cross-sectional	400	18.2	Low- Risk

The prevalence of unintended pregnancy varied significantly between studies included in this analysis, ranging from a reported high of 92.2% [22] to a reported low of 5.8% [21]. Furthermore, the sample sizes of these studies also showed significant heterogeneity, with the largest study including a sample size of 10,224 [47], while the smallest study included a sample size of 137 [35]. It is important to highlight that all studies included in this analysis underwent a rigorous assessment using the Joanna Briggs Institute (JBI) quality assessment checklist and were found to have a low risk of bias (Table 1).

### Meta-analysis

#### Prevalence of unintended pregnancy among contraceptive-user women in low- and middle-income countries

**Fig. 2:**
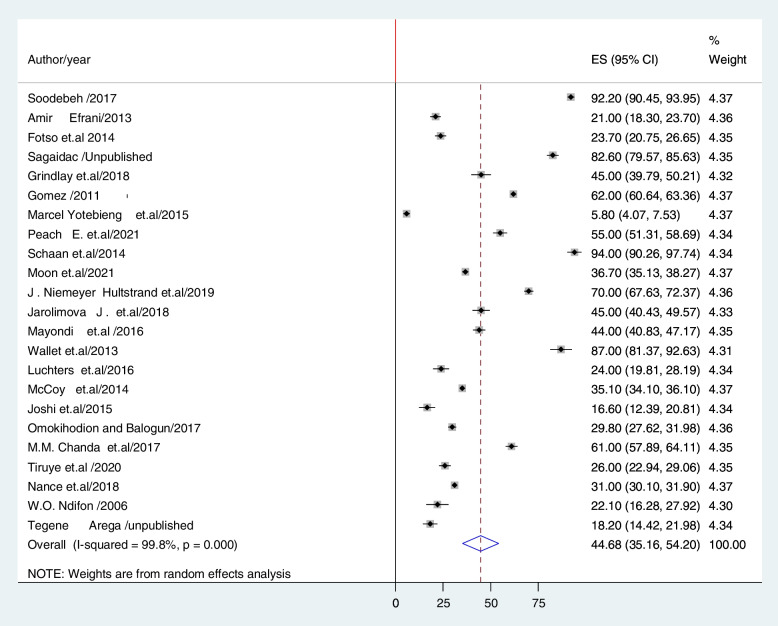
The pooled prevalence of unintended pregnancy among previous contraceptive user women in low- and middle-income countries

The overall estimate of unintended pregnancies among contraceptive users is shown using a forest plot (Fig. 2). The pooled prevalence of unintended pregnancy among contraceptive users in low- and middle-income countries was 44.68% (95% CI: 35.16–54.20; I2 = 99.7%, P < 0.001)., to the random effects model.

#### Source of heterogeneity

Leave-one-out sensitivity analysis

To determine the impact of individual studies on the overall prevalence of unintended pregnancy among contraceptive-using women, a sensitivity analysis using the leave-one-out method was carried out. One study at a time was excluded from this process. The results of the random effect model showed that none of the excluded studies had a statistically significant impact on the total estimate of unintended pregnancies (Table 2).

**Table 2:** Sensitivity analysis of unintended pregnancy among previous contraceptive user women in low—and middle-income countries

Study omitted	Estimate	95% confidence level
Soodebech	41.065685	34.012203—48.119164
Fotso et.al	44.073624	35.221188—52.92606
Sagaidac	42.629814	33.799519—51.46011
Grindlay et.al	43.146587	34.311317—51.981857
Gomez	42.401474	33.658314—51.144634
Marcel Yotebieng et.al	44.857098	36.602638—53.111557
Pearch E et.al	42.71167	33.868408—51.554932
Michelle M Schaan	41.201317	32.571857—49.830776
Mayondi et.al	43.18961	34.292522—52.086693
Moon et.al	43.509659	34.319794—52.699528
J. Niemayer Hultstrand et.al	42.05373	33.396263 – 50.711197
Jarolimova J.et.al	43.146355	34.300125—51.992584
Wallet et.al	41.341595	32.643204—50.039986
Luchters et.al	44.056171	35.228031—52.884315
McCoy et.al	43.581387	33.908459—53.254314
Joshi et.al	44.376541	35.5812—53.171883
Omokhodion and Balogun	43.80965	34.848541—52.77076
M.M.Chanda et.al	42.449173	33.632507—51.265835
Tiruye et.al	43.973183	35.109383—52.836987
Nance et.al	43.760494	34.141903 53.3790824
Tegegne Arega	44.309433	35.505627 53.113235
W.O.Ndifon	44.129772	35.319607 52.939934
Combined	44.68	35.163383 54.200444

##### Subgroup analysis

**Fig. 3:**
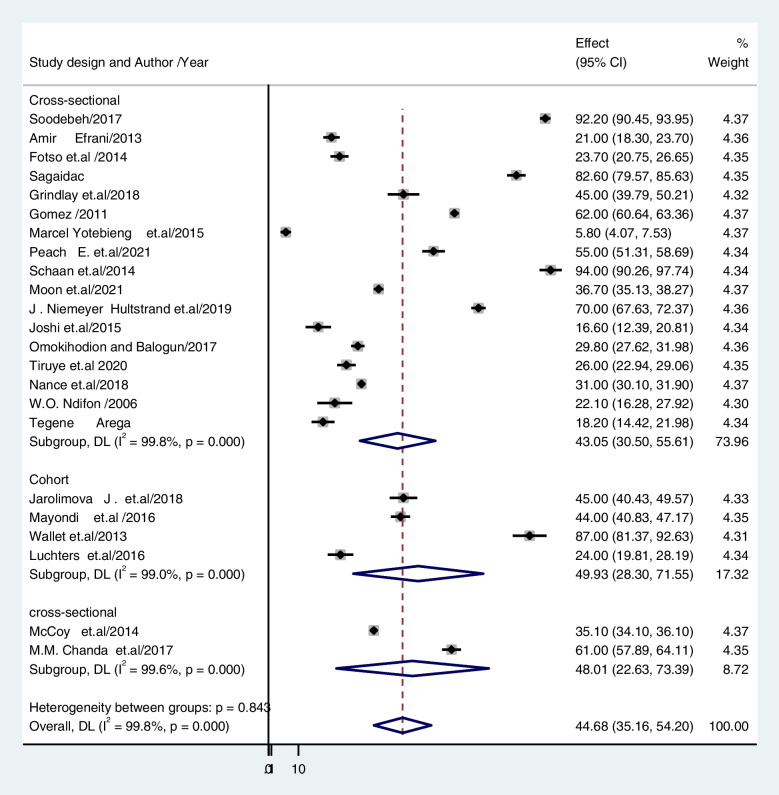
The pooled prevalence of unintended pregnancy among contraceptive user women in low- and middle-income countries based on study setting 2022

**Fig. 4:**
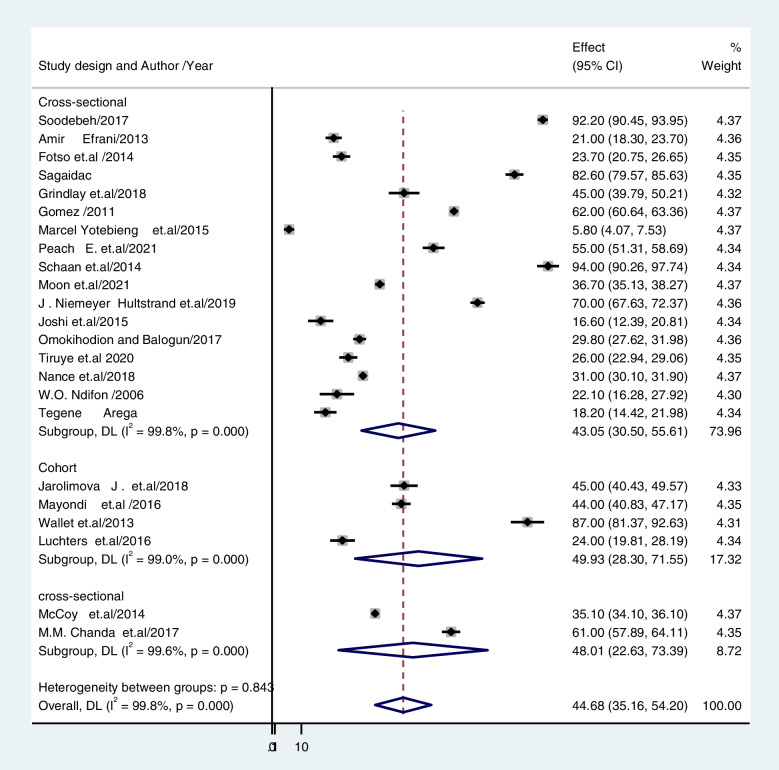
The pooled prevalence of unintended pregnancy among contraceptive-user women in low- and middle-income countries based on study design

**Fig. 5:**
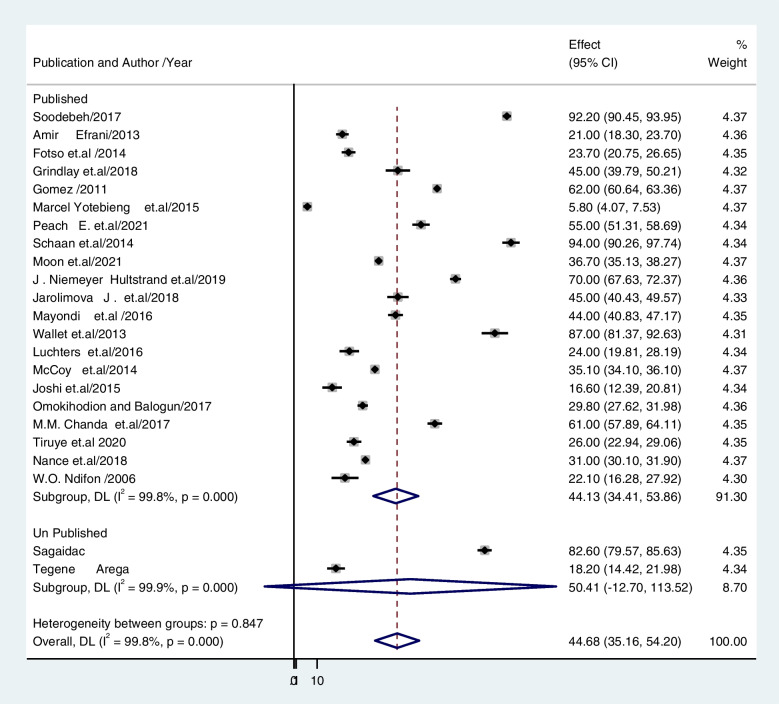
The pooled prevalence of unintended pregnancy among contraceptive-user women in low- and middle-income countries based on publication status

The subgroup analysis used in this study was based on heterogeneity. With a P value of less than 0.001, the Cochrane I^2^ statistic showed that there was significant heterogeneity at 99. 77%. As a result, a subgroup analysis was conducted using the study's setting, design, and status of publication (published vs. unpublished). The findings revealed that the prevalence of unintended pregnancy among contraceptive-user women was 34.47% (CI: 27.012, 41.933) in studies conducted in communities and 55.85% (CI: 33.364, 78.339) in studies conducted in institutions (Fig. 3). Regarding the study design, the prevalence of unintended pregnancy was 43. 58% for cross-sectional studies (CI: 32. 99, 54.173) and 49. 93% for cohort studies (CI: 28. 298, 71. 555) (Fig. 4). Based on publication, 43.74% of unintended pregnancies were published in articles, and 43.22% were not published (Fig. 5).

#### Publication bias

**Fig. 6:**
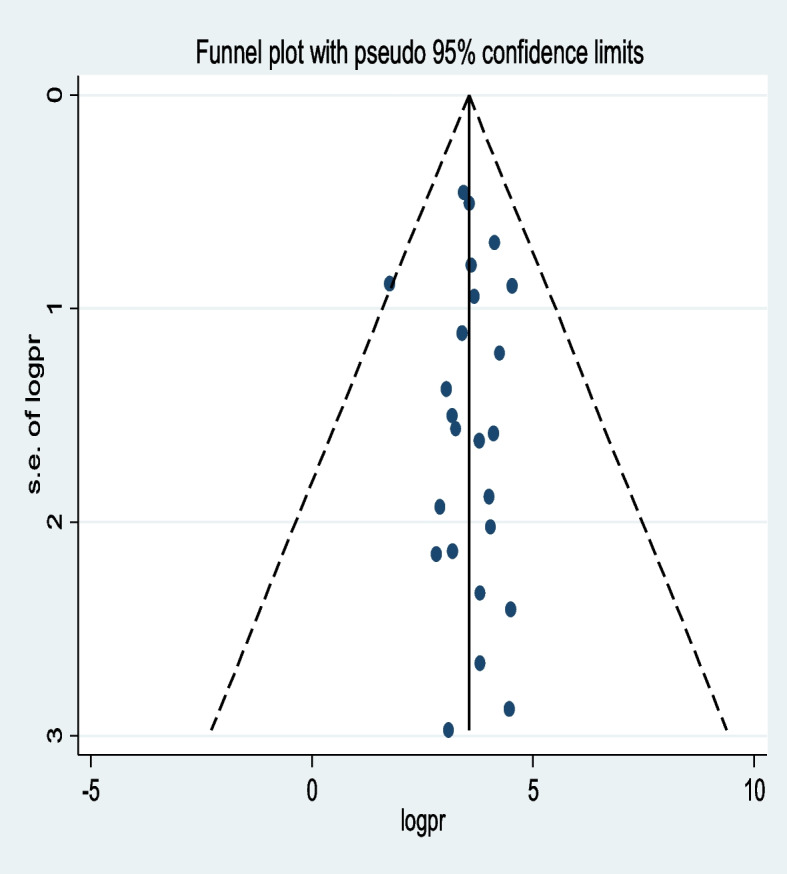
Funnel plot for the publication bias of unintended pregnancy among contraceptive-user women in low- and middle-income countries

The presence of publication bias was assessed using both subjective and objective methods. Subjectively, a funnel plot visualization was employed, while objectively, Egger's and Begg's tests were conducted (*P* < 0.05). The funnel plot analysis revealed a symmetrical distribution of studies (Fig. 6). Furthermore, the results of both Egger’s test (*P* = 0.834) and Begg’s test (*P* = 0.264) indicated a lack of evidence supporting the presence of publication bias in the included studies.

## Discussion

Based on the previous research findings, future development of sexual and reproductive health research is expected to focus predominantly on maternal health by 2030 [49]. This emphasis is driven by concerns about unintended pregnancy, which is widely recognized as a significant public health problem and imposes significant health, economic, and psychosocial burdens on both individuals and communities. Furthermore, unintended pregnancy can result in significant emotional distress for women, families, and society at large [50–52].

This study aims to provide an assessment of the overall prevalence of unplanned pregnancy in low- and middle-income countries through a systematic review and meta-analysis approach. By selecting and analyzing 23 studies that met the inclusion criteria, we were able to determine the overall prevalence of unintended pregnancy among women who had previously used contraceptive methods. The results of this systematic review and meta-analysis show that the pooled prevalence of unintended pregnancy among contraceptive users in low- and middle-income countries was 44.68% (95% CI: 35.16–54.20; I2 = 99.7%, *P* < 0.001).

In this review, we found a lower prevalence of unintended pregnancy compared to a study in 12 low- and middle-income countries. The above study reported a pooled prevalence of 86.8% [53]. The observed inconsistency between studies may be due to differences in study population and context. Specifically, the present research focused on women of childbearing age living in 23 low- and middle-income countries, while the first study targeted teenagers in 12 low- and middle-income countries. This disparity can be attributed to the fact that as women age, their desire and willingness to become pregnant tend to increase. Furthermore, another study conducted in 36 low- and middle-income countries found a 65% prevalence of contraceptive discontinuation among women with a current unintended pregnancy [54]. Nevertheless, this review is consistent with a study conducted in China among married women, which reported the prevalence rate to be 42.2% [55]. Similarly, a report by Bearak, J et al. conducted a global study that reported a prevalence rate of 45% [56].

On the other hand, the results of the present study indicate a higher prevalence of unintended pregnancy compared to a study conducted by Ahinkorah BO, which reported a prevalence of 22.4% in selected sub-Saharan African countries [57]. The observed disparity may be attributed to differences in the demographic composition of study participants, sample size, and the contextual setting of the study. More specifically, the present research included women of childbearing age from 23 nations, while Ahinkorah BO's study focused exclusively on young women living in the ten sub-Saharan African countries characterized by the highest fertility rates. Moreover, the prevalence rate observed in this study exceeded the results of previous demography and health survey studies conducted in Bangladesh [58], which reported the rate to be 24.3%. Additionally, the current prevalence rate was higher than the rate reported in a thorough systematic review and meta-analysis done in Ethiopia, which reported a prevalence rate of 28% [59]. Additionally, it was higher than the prevalence rate of 26.46% which was noted in 61 Demographic and Health Surveys (DHS) conducted in low- and middle-income countries (LMICs) [60]. The discrepancy may result from variations in the number of countries examined, the population sizes of those countries, the health system of each country, and the sample sizes employed.

Finally, this review aims to provide important data for stakeholders, including policymakers, healthcare providers, scientific community to facilitate the development of effective strategies and treatments for the management and control of unplanned pregnancies in low- and middle-income countries.

## Strengths and limitations of the study

We conducted a systematic literature review and included research based on clearly defined criteria. We only examined English-language publications. Preprinted articles that had not yet been peer-reviewed were also included. The results of these studies may therefore change in subsequent studies, and methodological biases may occur.

## Conclusion

The overall prevalence of unintended pregnancy was high among women using contraceptives in low- and middle-income countries. In addition, the pooled prevalence of unintended pregnancy differed based on the study setting, publication, and study design. Accordingly, it is better to pay attention to the prevention strategies of unintended pregnancy, such as information and education accessibility and contraceptive utilization.

### Supplementary Information

**Additional file 1:** **Table 1.** PRISMA checklist.

**Additional file 2:** **Table 2.** Methodological quality assessment tool for the included studies of unintended pregnancy.

**Additional file 3:** **Table 3.** Risk of bias assessment for the included studies of unintended pregnancy among the previous contraceptive.

## Data Availability

All relevant data are within the Manuscript and its Supporting Information files.
