# Correction: Unintended pregnancy and contraceptive use among women in low- and middle-income countries: systematic review and meta-analysis

**DOI:** 10.1186/s40834-023-00258-4

**Published:** 2023-12-07

**Authors:** Kelemu Abebe Gelaw, Yibeltal Assefa Atalay, Natnael Atnafu Gebeyehu

**Affiliations:** 1 School of Midwifery, College of Medicine and Health Sciences, Wolaita Sodo University, Wolaita Sodo, Ethiopia; 2 School of Public Health, College of Health Science and Medicine, Wolaita Sodo University, Wolaita Sodo, Ethiopia

**Correction:** ***Contracept Reprod Med*** **8, 55 (2023)**

10.1186/s40834-023-00255-7

Following publication of the original article [1], the authors reported an error that the author name Yibeltal Assefa Atalay was incorrectly written as Yibeletal Assefa Atalay.

The original article [1] has been updated.

## References

[1] Gelaw KA, Atalay YA, Gebeyehu NA (2023). Unintended pregnancy and contraceptive use among women in low- and middle-income countries: systematic review and meta-analysis. Contracept Reprod Med.

