# Supplementary material for: Unintended pregnancy and contraceptive use among women in low- and middle-income countries: systematic review and meta-analysis
Source: Contracept Reprod Med. 2023 Nov 23;8:55. doi: 10.1186/s40834-023-00255-7 (PMC10666441; doi:10.1186/s40834-023-00255-7)
Supplement: Supplementary file 2 — Additional file 2: Table 2. Methodological quality assessment tool for the included studies of unintended pregnancy. [file 40834_2023_255_MOESM2_ESM.docx]

Table 2. Methodological quality assessment tool for the included studies of unintended pregnancy

| Item | \| Clearly defined inclusion criteria \| \| --- \| | \| Describing the study settings and participants \| \| --- \| | \| Valid and reliable exposure measurement \| \| --- \| | \| Objective and standard criteria for measurement \| \| --- \| | \| Identified confounder \| \| --- \| | \| Strategies to deal with confounder \| \| --- \| | \| Valid and reliable outcome measurement \| \| --- \| | \|  \| \| --- \|   Appropriate  statistical analysis | \| **yes**  **(%)** \| \| --- \| |
| --- | --- | --- | --- | --- | --- | --- | --- | --- | --- | --- | --- | --- | --- | --- | --- | --- | --- | --- |
| Soodebech | Yes | Yes | Yes | Yes | Yes | No | Yes | No | 6/8=75 |
| Amir Erfani | Yes | Yes | Yes | Yes | Yes | Yes | No | Yes | 7/8=87.5 |
| Fotso et.al | Yes | Yes | No | Yes | Yes | Yes | No | Yes | 6/8=75 |
| Sagaidac et.al | Yes | No | Yes | Yes | No | No | Yes | Yes | 5/8=62.5 |
| Grindlay et.al | Yes | Yes | No | Yes | No | No | Yes | Yes | 5/8=62.5 |
| Gomez | Yes | Yes | No | Yes | Yes | Yes | No | Yes | 6/8=75 |
| Marcel Yotebieng  Et.al | Yes | Yes | Yes | Yes | Yes | Yes | No | Yes | 7/8=87.5 |
| Pearch E .et.al | Yes | Yes | No | Yes | Yes | Yes | No | Yes | 6/8=75 |
| Sachaan et.al | Yes | Yes | Yes | Yes | Yes | Yes | No | Yes | 7/8=87.5 |
| J.Niemayer Hultstrand et.al | Yes | Yes | No | Yes | Yes | Yes | No | Yes | 6/8=75 |
| Moon et.al | Yes | Yes | No | Yes | Yes | Yes | No | Yes | 6/8=75 |
| McCoy et.al | Yes | Yes | No | Yes | Yes | Yes | No | Yes | 6/8=75 |
| Joshi et.al | Yes | Yes | No | Yes | No | Yes | Yes | No | 5/8=62.5 |
| Omokhodion  & Balogun | Yes | Yes | No | Yes | Yes | Yes | No | Yes | 6/8=75 |
| M. M. Chanda et.al | Yes | Yes | Yes | Yes | Yes | Yes | No | Yes | 7/8=87.5 |
| Tiruye et.al | Yes | Yes | Yes | Yes | Yes | Yes | No | Yes | 7/8=87.5 |
| Nance et.al | Yes | Yes | No | Yes | No | Yes | Yes | No | 5/8=62.5 |
| W. O. Nidfon | Yes | Yes | Yes | Yes | Yes | Yes | No | Yes | 7/8=87.5 |
| Tegene Arega | Yes | Yes | Yes | Yes | No | Yes | No | Yes | 6/8=75 |
| Mayondi et. al | Yes | Yes | No | Yes | Yes | Yes | Yes | Yes | 7/8=87.5 |
| Jarolimova  J.et.al | Yes | No | Yes | Yes | No | Yes | Yes | Yes | 6/8=75 |
| Luchters et.al | Yes | Yes | No | Yes | Yes | Yes | Yes | Yes | 7/8=87.5 |
| Wallet et.al | Yes | Yes | No | Yes | Yes | yes | Yes | No | 6/8=75 |
