# Supplementary material for: Unintended pregnancy and contraceptive use among women in low- and middle-income countries: systematic review and meta-analysis
Source: Contracept Reprod Med. 2023 Nov 23;8:55. doi: 10.1186/s40834-023-00255-7 (PMC10666441; doi:10.1186/s40834-023-00255-7)
Supplement: Supplementary file 3 — Additional file 3: Table 3. Risk of bias assessment for the included studies of unintended pregnancy among the previous contraceptive. [file 40834_2023_255_MOESM3_ESM.docx]

Table 3. Risk of bias assessment for the included studies of unintended pregnancy among the previous contraceptive

| **Item** | **External validity** | | | | | | | **Internal validity** | | | | | | | | | | | |  | | |
| --- | --- | --- | --- | --- | --- | --- | --- | --- | --- | --- | --- | --- | --- | --- | --- | --- | --- | --- | --- | --- | --- | --- |
| \|  \| \| --- \|   **For cross-sectional studies** | **Representativeness of the target population** | | **Representativeness of the sampling frame** | **Random sampling or census** | | **Minimal response bias** | | **Data were collected directly** | | **Acceptable case definition used in the study**   \|  \| \| --- \| | | **Valid and reliable measurement** | | **The same mode of data collection for all study subject** | | **Appropriate length of prevalence period for parameter of interest** | | **Appropriate numerators and denominators of interest** | | No of yes | | **Summary of risk of bias** |
| Soodebech   \|  \| \| --- \| | Yes | | Yes | Yes | | Yes | | No | | No | | Yes | | Yes | | Yes | | Yes | | 8 | | Loiw risk |
| Amir Erfani | Yes | | Yes | No | | Yes | | Yes | | No | | Yes | | Yes | | Yes | | Yes | | 8 | | Low-risk |
| Fotso et.al | Yes | | Yes | No | | Yes | | Yes | | Yes | | Yes | | Yes | | Yes | | Yes | | 9 | | Low –risk |
| Sagaidac et.al   \|  \| \| --- \| | Yes | | Yes | No | | Yes | | Yes | | Yes | | Yes | | Yes | | Yes | | Yes | | 9 | | Low-risk |
| Grindlay et.al | Yes | | Yes | Yes | | Yes | | Yes | | No | | Yes | | Yes | | Yes | | Yes | | 9 | | Low-risk |
| Gomez | Yes | | Yes | No | | Yes | | Yes | | Yes | | Yes | | Yes | | Yes | | Yes | | 9 | | Low-risk |
| Marcel Yotebieng  Et.al   \|  \| \| --- \| | Yes | | Yes | Yes | | Yes | | No | | No | | Yes | | Yes | | Yes | | Yes | | 8 | | Low-risk |
| Pearch E .et.al | Yes | | Yes | No | | Yes | | Yes | | Yes | | Yes | | No | | Yes | | Yes | | 8 | | Low-risk |
| Sachaan et.al   \|  \| \| --- \| | Yes | | Yes | Yes | | Yes | | No | | Yes | | Yes | | No | | Yes | | Yes | | 8 | | Low-risk |
| J.Niemayer Hultstrand et.al | Yes | | Yes | Yes | | Yes | | No | | No | | Yes | | Yes | | Yes | | Yes | | 8 | | Low-risk |
| Moon et.al | Yes | | Yes | No | | Yes | | Yes | | Yes | | Yes | | Yes | | Yes | | Yes | | 9 | | Low-risk |
| McCoy et.al | Yes | | Yes | Yes | | No | | Yes | | No | | Yes | | Yes | | Yes | | Yes | | 8 | | Low-risk |
| Joshi et.al | Yes | | Yes | Yes | | Yes | | Yes | | Yes | | Yes | | No | | Yes | | Yes | | 9 | | Low-risk |
| Omokhodion  & Balogun | Yes | | Yes | Yes | | Yes | | Yes | | Yes | | No | | Yes | | Yes | | Yes | | 8 | | Low-risk |
| M. M. Chanda et.al | Yes | | Yes | No | | Yes | | Yes | | Yes | | Yes | | Yes | | Yes | | Yes | | 9 | | Low risk |
| Tiruye et.al | Yes | | Yes | Yes | | Yes | | Yes | | Yes | | Yes | | Yes | | No | | No | | 8 | | Low-risk |
| Nance et. | Yes | | Yes | Yes | | Yes | | Yes | | Yes | | Yes | | No | | No | | Yes | | 8 | | Low-risk |
| W. O.Nidfon | Yes | | Yes | Yes | | Yes | | Yes | | Yes | | Yes | | Yes | | Yes | | No | | 9 | | Low-risk |
| Tegene Arega | Yes | | Yes | No | | Yes | | Yes | | Yes | | Yes | | No | | Yes | | Yes | | 8 | | Low-risk |
| **For prospective cohort** | **Criteria** | | | | | | | | | | | | | | | | | | | | | |
| **Item** | **Two groups are similar and recruited from the same population** | **Similar measurement of exposure both for exposed and**  **unexposed groups** | | | **Valid and reliable measurement of exposure** | | **Identifying confounders** | | **Strategies to deal with confounders** | | **Groups are free of the outcomes at the beginning** | | **Valid and reliable measurement of outcomes** | | **Long enough follow-up**  **time for the occurrence of outcomes** | | **Complete follow-up time** | | **Strategies to address lost follow-up** | | **Percentage of yes (%)** | **Summary of risk of bias** |
| Myondi et.al | Yes | Yes | | | No | | Yes | | Yes | | Yes | | Yes | | No | | Yes | | Yes | | 8 | Low-risk |
| Jarolinova .J et.al | Yes | Yes | | | Yes | | No | | Yes | | No | | Yes | | Yes | | Yes | | Yes | | 8 | Low-risk |
| Luchters et.al | Yes | Yes | | | Yes | | No | | Yes | | Yes | | Yes | | Yes | | Yes | | Yes | | 9 | Low-risk |
| Wallet et.al | Yes | Yes | | | Yes | | No | | Yes | | No | | Yes | | Yes | | Yes | | Yes | | 8 | Low-risk |
